# Dose-Related Antihypertensive Properties and the Corresponding Mechanisms of a Chicken Foot Hydrolysate in Hypertensive Rats

**DOI:** 10.3390/nu10091295

**Published:** 2018-09-12

**Authors:** Anna Mas-Capdevila, Zara Pons, Amaya Aleixandre, Francisca I. Bravo, Begoña Muguerza

**Affiliations:** 1 Nutrigenomics Research Group, Department of Biochemistry and Biotechnology, Universitat Rovira i Virgili, 43007 Tarragona, Spain; anna.mas@urv.cat (A.M.-C.); zara.pons@urv.cat (Z.P.); begona.muguerza@urv.cat (B.M.); 2 Department of Pharmacology, School of Medicine, Universidad Complutense de Madrid, 28040 Madrid, Spain; amaya@med.ucm.es; 3 Technological Unit of Nutrition and Health, EURECAT-Technology Centre of Catalonia, 43204 Reus, Spain

**Keywords:** hypertension, protein hydrolysate, angiotensin-converting enzyme, ACE-inhibitory activity, endothelial dysfunction, bioactive peptides

## Abstract

The antihypertensive properties of different doses of a chicken foot hydrolysate, Hpp11 and the mechanisms involved in this effect were investigated. Spontaneously hypertensive rats (SHR) were administered water, Captopril (50 mg/kg) or Hpp11 at different doses (25, 55 and 85 mg/kg), and the systolic blood pressure (SBP) was recorded. The SBP of normotensive Wistar-Kyoto (WKY) rats administered water or Hpp11 was also recorded. Additionally, plasmatic angiotensin-converting enzyme (ACE) activity was determined in the SHR administered Hpp11. Moreover, the relaxation caused by Hpp11 in isolated aortic rings from Sprague-Dawley rats was evaluated. Hpp11 exhibited antihypertensive activity at doses of 55 and 85 mg/kg, with maximum activity 6 h post-administration. At this time, no differences were found between these doses and Captopril. Initial SBP values of 55 and 85 mg/kg were recovered 24 or 8 h post-administration, respectively, 55 mg/kg being the most effective dose. At this dose, a reduction in the plasmatic ACE activity in the SHR was found. However, Hpp11 did not relax the aortic ring preparations. Therefore, ACE inhibition could be the mechanism underlying Hpp11 antihypertensive effect. Remarkably, Hpp11 did not modify SBP in WKY rats, showing that the decreased SBP effect is specific to the hypertensive state.

## 1. Introduction

Cardiovascular diseases (CVD) are the leading cause of mortality in Europe, and hypertension (HTN) is one of the main CVD risk factors [1]. In this sense, the lowering of blood pressure (BP) through behavioural and pharmacological interventions has been showed to remarkably improve CVD [2]. Currently, one of the most popular pharmacologic therapies to treat HTN is based on the use of angiotensin-converting enzyme (ACE) inhibitors such as Captopril or Enalapril [3]. ACE is the key enzyme of the renin-angiotensin-aldosterone system (RAAS), which is one of the most important systems in the regulation of blood volume and systemic vascular resistance. ACE hydrolyses the decapeptide angiotensin I (Ang I) to the octapeptide angiotensin II (Ang II), which is a potent vasoconstrictor but also breaks down bradykinin, a vasodilator [4]. Although pharmacological ACE inhibitors are widely used to decrease BP, some undesirable side effects have been described for these drugs, such as angioedema, dry cough, disturbance in taste, and skin reactions, among others [5]. In consequence, the study of natural bioactive compounds has received great attention, and their use has been considered a good strategy to decrease the risk of HTN [6].

Specifically, the hydrolysis of food proteins is considered a potential source of peptides with ACE inhibitory activities and/or antihypertensive effects [7,8,9,10]. Different protein sources have been reported to release ACE inhibitor peptides, such as milk and egg [11], fish [12,13] or meat [14], among others. Considering this, the interest in proteins derived from food by-products as sources of ACE inhibitor peptides has increased. The use of food by-products allows the reuse of waste materials, making the food and agricultural industries more environmentally friendly [15,16,17]. In this regard, our group has used chicken feet, a poultry industry by-product, to obtain hydrolysates that present ACE inhibitory (ACEI) activity [18]. Chicken feet is considered a by-product in Spain and in most countries in Europe, and since a few years ago, the conversion of animal by-products to feed and certain legislations in some countries disallows indiscriminate dumping or landfilling of animal wastes [19]. Thus, this chicken by-product represents environmental and economic problems for meat processors if they are not correctly treated. Considering this, the chicken industry considered that it is no longer practical to discard by-products and wastes, especially when a significant amount of valuable raw materials have a strong economic potential like the production of new products and functional ingredients with a significant added-value [20]. It is known that chicken proteins, especially chicken collagen, has been demonstrated to be a source of bioactive peptides, able to inhibit ACE and exhibit antihypertensive activity [21,22,23]. Nevertheless, the in vitro ACEI activity and in vivo antihypertensive effects of protein hydrolysates are not always correlated, since the physiological transformations during digestion determine the bioavailability of these peptides and, as a result, their bioactivity. Moreover, it has also been demonstrated that the in vivo ACEI activity is not the only mechanism underlying the antihypertensive effects of some bioactive peptides. In this sense, certain food bioactive peptides have shown direct effects on relaxation in vascular smooth muscle [24].

In a previous study, we observed that the administration of 5 mL/kg bw (body weight) of a chicken foot hydrolysate, Hpp11, exhibited an antihypertensive effect in spontaneously hypertensive rats (SHR), which is considered to be a model for human essential HTN [25]. Thus, considering that 100 mg/kg bw dose of other protein hydrolysates showed substantial antihypertensive effects [26,27,28], doses lower than the antihypertensive demonstrated dose of 100 mg/kg bw; 85, 55 and 25 mg/kg bw of Hpp11, were administered to SHR. Therefore, the aims of the present study were to evaluate the most effective Hpp11 dose to obtain a significant antihypertensive effect in SHR and to investigate the mechanisms underlying the Hpp11 antihypertensive effect. Moreover, the effect of Hpp11 on the arterial SBP of Wistar-Kyoto (WKY) rats, the normotensive control for SHR, was also studied to rule out a potential hypotensive effect of this hydrolysate.

## 2. Materials and Methods

### 2.1. Chemicals and Reagents

Chicken feet from *Gallus gallus domesticus* were provided by a local farm (Granja Gaià, La Riera de Gaià, Spain). Protamex^®^ (Novozymes, Bagsværd, Denmark) (EC 3.4.21.62 and 3.4.24.28, 1.5 AU/g from *Bacillus licheniformis* and *Bacillus amyloliquefaciens*), was kindly provided by Novozymes (Bagsværd, Denmark). ACE (angiotensin-converting enzyme, EC 3.4.15.1), *N*-Hippuryl-His-Leu (Hip-His-Leu), Captopril (PubChem CID: 44093) were purchased from Santa Cruz Biotechnology (Dallas, TX, USA) and o-aminobenzoylglicil-p-nitrofenilalanilprolina (o-Abz-Gly-p-Phe(NO_2_)-Pro-OH, PubChem CID: 128860) was provided by Bachem Feinchemikalien (Bubendorf, Switzerland). Acetylcholine (PubChem CID: 187), methoxamine hydrochloride (PubChem CID: 6081) and heparin heparin (PubChem CID: 772) were purchased from Sigma-Aldrich (Madrid, Spain). All other chemical solvents used were of analytical grade.

### 2.2. Chicken Foot Hydrolysate Hpp11: Obtainment and Characterisation

Chicken feet were mechanically disrupted, and sieves were utilized to obtain the protein hydrolysate, Hpp11 [18]. Protein powder with a size ≤2 mm was suspended in distilled water (20 mg/mL, *w*/*v*) and incubated for 1.5 h in a water bath set at 100 °C at 100 rpm. Subsequently, an enzymatic solution, Protamex^®^, was added at a final concentration of 2.67 µg/mL (enzyme/substrate ratio, 0.4 AU/g protein). Hydrolysis was carried out at 50 °C for 2 h at pH 7.0 in a MaxQ Orbital Shaker Thermo Scientific (Thermo Fisher Scientific, Waltham, MA, USA). At the end of the reaction, the enzyme was heat inactivated (80 °C, 10 min) in a water bath. Then, hydrolysate was centrifuged at 10,000× *g* for 20 min at 4 °C, and the supernatant was filtered through a 0.45 μm membrane, collected and lyophilized. Hpp11 was reconstituted in water to carry out the following experiments.

Hpp11 was characterized before its administration to SHR. Hpp11 protein content was estimated by the determination of total nitrogen compounds content of Hpp11 by the Kjeldahl method, multiplying the determined nitrogen content by 6.25 and the humidity determination was carried out following the AOAC official methods [29]. The degree of hydrolysis was determined by the TNBS method according to Adler-Nissen (1979) [30], in which free α-amino groups were determined. The Hpp11 ACEI activity was determined according to Quirós et al. [31]. The fluorescence measurements were performed after 30 min in a multi-scan microplate fluorimeter (FLUOstar optima, BMG Labtech, Offeuburg, Germany). The excitation and emission wavelengths were 360 and 400 nm, respectively. The software used to process the data was FLUOstar control (version 1.32 R2, BMG Labtech, Offeuburg, Germany).

The inhibition pattern of Hpp11 on the ACE substrate o-Abz-Gly-p-Phe(NO_2_)-Pro-OH was assayed at the following concentrations: 7.2, 3.6, 1.8, 0.9, 0.45, 0.23 and 0 mM. The inhibition kinetics of ACE in the presence of Hpp11 was determined by Lineweaver–Burk plots [30].

All the analyses were performed in triplicate.

### 2.3. Experimental Procedure in the SHR and WKY Rats

Male SHR and WKY rats (17–20-week-old, weighing 300–350 g) were obtained from Charles River Laboratories España S.A. (Barcelona, Spain). The animals were housed at a temperature of 23 °C with 12 h light/dark cycles and consumed tap water and a standard diet (A04 Panlab, Barcelona, Spain) ad libitum during the experiments.

Different doses of the hydrolysate (25, 55 and 85 mg/kg bw) or a single dose of Hpp11 (55 mg/kg bw) were administered by gastric intubation to SHR or WKY rats, respectively, between 9 and 10 am. Tap water was used as a negative control for the SHR and WKY rats, and 50 mg/kg Captopril dissolved in tap water was given as a positive control to the SHR. The total volume of water, Captopril or Hpp11 orally administered to the rats was between 1.5 and 2 mL.

The systolic blood pressure (SBP) was recorded in the rats by the tail-cuff method [32] before and 2, 4, 6, 8, 24 and 48 h post-administration. Before the measurement, the animals were kept at 38 °C for 10 min in order to detect the pulsations of the tail artery. Changes in the SBP were expressed as the differences between the mean values of these variables before and after the administration of the treatment. To minimize stress-induced variations in BP, all measurements were taken by the same person, in the same peaceful environment. Moreover, before starting the experiments, we established a 2-week training period for the rats to become accustomed to the procedure. Data are expressed as the mean values ± standard error of the means (SEM) for a minimum of six experiments.

Additionally, twelve 20–23-week-old SHR weighing 350–380 g were administered Hpp11 at 55 mg/kg bw or water to determine the plasmatic ACE activity. The Hpp11 and water were orally administered by gastric intubation between 9 and 10 am. Blood samples were collected at 6 h post-administration via the saphenous vein using heparin vials. The samples were centrifuged at 2000× *g* for 15 min at 4 °C to obtain plasma. The procedure that was used to determine the plasmatic ACE activity is described below.

### 2.4. Determination of the Plasmatic ACE Activity

The plasmatic ACE activity was performed by a fluorometric method reported by Miguel et al. [28]. The measurements were performed in a multi-scan microplate fluorimeter (FLUOstar optima, BMG Labtech) at 37 °C and 350 nm excitation with 520 nm emission filters. ACE at different concentrations was added to each plate to obtain a calibration curve. ACE activity was expressed as the mean ± SEM mU ACE/mL of plasma for at least three replicates.

### 2.5. Experiments in Aorta Rings

Male 17–22-week-old, non-treated Sprague-Dawley (SD) rats weighing 250–300 g were sacrificed by decapitation. The thoracic aorta was excised from the animal’s thorax, and excess fat and connective tissue were removed. To obtain the aorta preparations, the tissue was placed in a dissecting dish containing Krebs-Henseleit solution (NaCl, 118 mM: KCl, 4.7 mM; CaCl_2_, 2.5 mM; KH_2_PO_4_, 1.2 mM; MgSO_4_, 1.2 mM; NaHCO_3_, 25 mM; and glucose, 10.0 mM) and cut into 3–4 mm rings. The aorta rings were mounted between two steel hooks in organ baths containing Krebs-Henseleit solution at 37 °C and continuously bubbled with a 95% O_2_ and 5% CO_2_ mixture, which gave a pH of 7.4. An optimal tension of 2 g was applied to all the aortic rings and adjusted every 15 min during the 60–90 min equilibration period, before adding the assayed compounds. The isometric tension was recorded by using an isometric force displacement transducer connected to an acquisition system (Protos 5, Panlab, Barcelona, Spain). After the equilibration period, 80 mM KCl was added to verify their functionality, and when the contraction had reached the steady state (approximately 15 min after the administration), the preparations were washed until the basal tension was recovered. The rings were then exposed to 10^−5^ M methoxamine, and when the contraction had reached the steady state, 10 µL Hpp11 was added to the organ bath at cumulative doses to reach concentrations between 0.01 mg/mL and 5 mg/mL. Water (10 µL) was used as negative control. The relaxant responses were expressed as a percentage of the pre-contraction induced by methoxamine, which was considered 100 percent. Results are expressed as the means ± SEM for at least eight experiments using aorta rings extracted from different animals. Concentration–response curves were fitted to the logistic equation, and statistical analysis was performed to compare concentration–response curves.

All the animal protocols followed in this study were approved by the Bioethical Committee of the Universitat Rovira i Virgili (European Comission Directive 86/609) and the Spanish Royal Decree 223/1988.

### 2.6. Statistical Analysis

The results are expressed as the mean ± SEM. Differences between the Hpp11 doses in the SHR were analysed by a two-way analysis of variance (ANOVA), and the Hpp11 effect on the WKY rats was analysed by a one-way analysis of variance (one-way ANOVA). To analyse differences between multiple independent groups, one-way analysis of variance followed by Tukey’s or Dunnet’s T3 post hoc test were used when required. The plasmatic ACE results were analysed by Student’s *t*-test. Differences between concentration–response curves were analysed by two-way analysis of variance (two-way ANOVA). All the analyses were performed using IBM SPSS Statistics (SPSS, Chicago, IL, USA). Outliers were determined by using Grubbs’ test. Differences between groups were considered significant when *p* < 0.05.

## 3. Results

### 3.1. Chicken Foot Hydrolysate Hpp11

Chicken foot hydrolysate, Hpp11, was characterized before the in vivo experiments. According to ACE inhibition, it was observed that the IC_50_ value (concentration of the hydrolysate needed to inhibit 50% of the original ACE activity) of Hpp11 was 0.027 mg/mL. Table 1 shows the results of the Hpp11 determination of protein content, expressed as the total nitrogen compounds content, the humidity, the ash content, the degree of hydrolysis and the ACE inhibition as percentage.

### 3.2. Hpp11 In Vitro Inhibition Pattern on ACE

**Figure 1:**
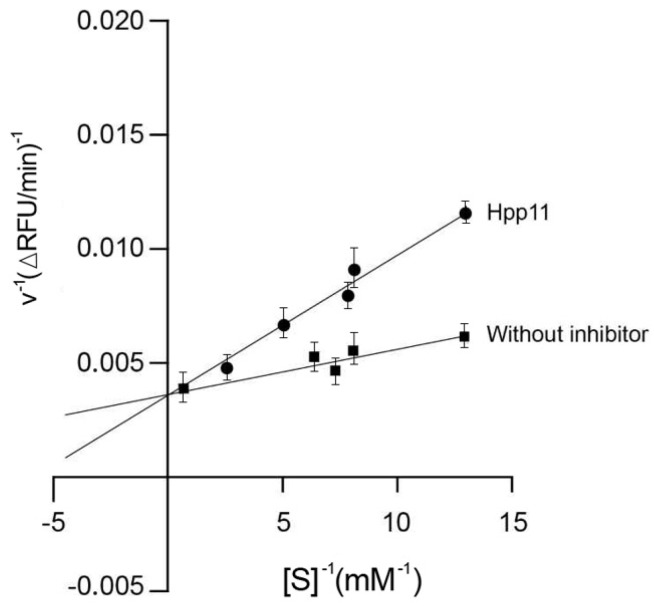
Lineweaver-Burk plot of angiotensin-converting enzyme (ACE) inhibition by chicken foot hydolysate Hpp11 and the control (without inhibitor). The Hpp11 effects at varying concentrations of ACE substrate (0–7.2 mM).

Figure 1 shows the Lineweaver-Burk plot of ACE activity in presence of Hpp11. Considering that the Lineweaver-Burk plot obtained by changing the substrate concentration intersects with the *y*-axis, the inhibition of ACE by Hpp11 corresponds to competitive inhibition.

### 3.3. Effect of Different Doses of Hpp11 on Blood Pressure in Hypertensive and Normotensive Rats

**Figure 2:**
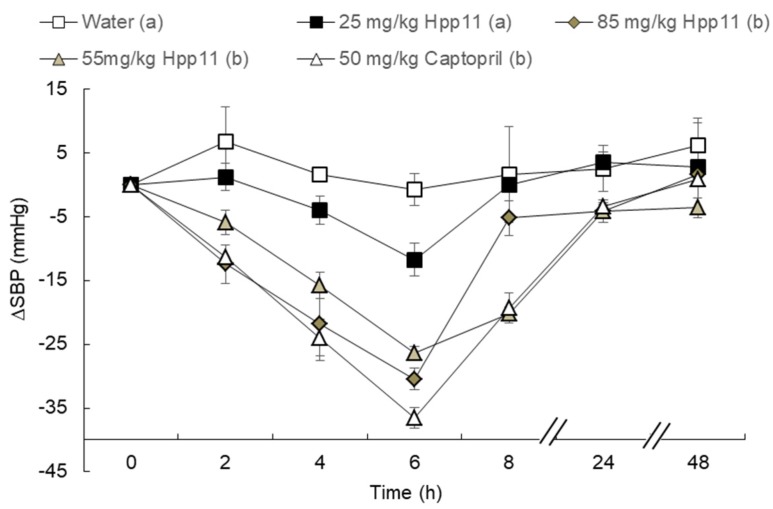
Decrease in the systolic blood pressure (SBP) in spontaneously hypertensive rats after the administration of water, Captopril (50 mg/kg bw) or different doses of chicken foot hydrolysate Hpp11: 25 mg/kg bw, 55 mg/kg bw and 85 mg/kg bw. Data are expressed as the mean ± SEM. All of the experimental groups include a minimum of six animals. Different letters represent significant differences (*p* < 0.05). *p* was estimated by two-way ANOVA.

Figure 2 shows the effect of three different doses of Hpp11 in SHR. Initial values of the SBP in the SHR were 195.9 ± 3.15 mmHg. As expected, the rats that only received water did not change their SBP values. In contrast, administration of Captopril (50 mg/kg bw) caused a clear decrease in the SBP, reaching the maximum decrease at 6 h post-administration. Regarding the hydrolysate, oral administration of 25 mg/kg bw did not produce an antihypertensive effect in the SHR. However, Hpp11 at 55 or 85 mg/kg bw resulted in a significant decrease in the SBP, reaching the maximum decrease at 6 h post-administration (−26.33 ± 2.1 and −30.45 ± 1.65 mmHg, respectively). At this time, the SBP decreases produced by both doses were similar to the decreases caused by Captopril. In fact, no significant differences between the decrease in BP produced by both Hpp11 doses was observed; however, the 55 mg/kg bw dose produced a more sustained antihypertensive effect than 85 mg/kg bw, showing a similar behaviour when compared to Captopril. In this sense, the SBP initial values were recovered 24 or 8 h post-administration at 55 and 85 mg/kg bw, respectively.

**Figure 3:**
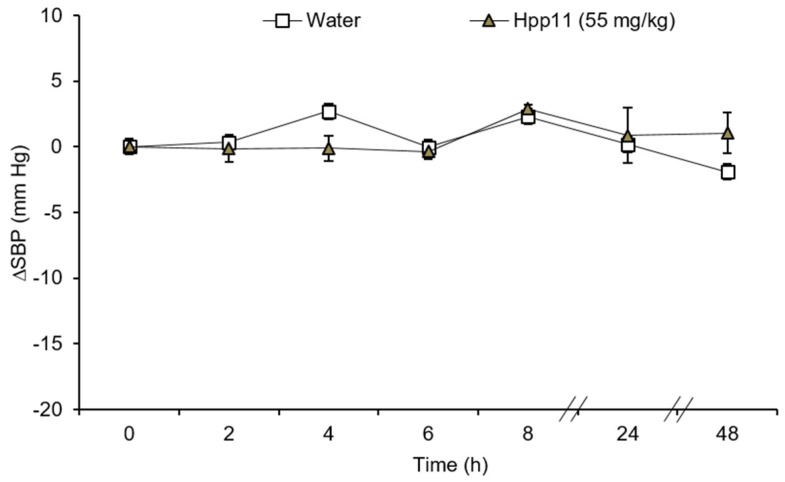
Decrease in the systolic blood pressure (SBP) in Wistar–Kyoto rats after administration of water or 55 mg/kg bw of chicken foot hydrolysate Hpp11. Data are expressed as the mean ± SEM. Both experimental groups have a minimum of six animals. No significant differences were observed.

In addition, oral administration of Hpp11 at a single dose of 55 mg/kg bw did not modify the arterial SBP in the normotensive WKY rats during the experiment (Figure 3). In fact, the SBP from the treated group showed similar values as the group administered with water.

### 3.4. Mechanisms Involved in the Antihypertensive Effect of Hpp11

**Figure 4:**
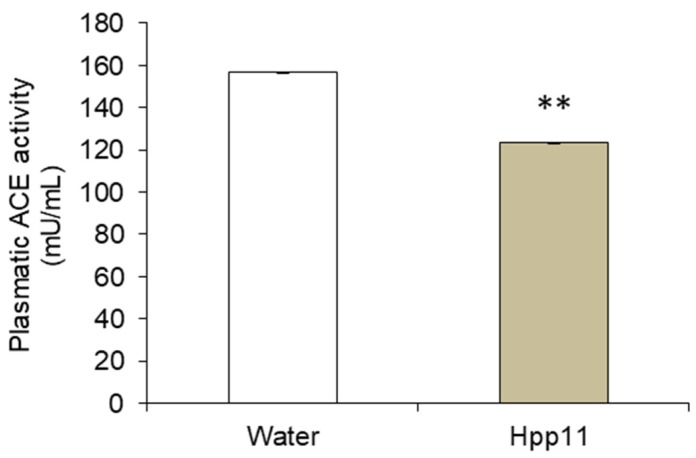
The plasmatic angiotensin-converting enzyme activity (ACE) in spontaneously hypertensive rats, 6 h after administration of 55 mg/kg chicken foot hydrolysate Hpp11 or water. Data are expressed as the mean ± SEM. The experimental groups include a minimum of six animals. The asterisks indicate differences between groups at *p* < 0.01 (**). *p* was calculated by Student’s *t*-test.

The plasmatic ACE activity was measured in the rats administered 55 mg/kg bw of Hpp11 or water, 6 h post-administration. A reduction of 21% in the plasmatic ACE activity was found in the group administered Hpp11, being significantly lower than plasmatic ACE activity presented by the group administered water (Figure 4).

**Figure 5:**
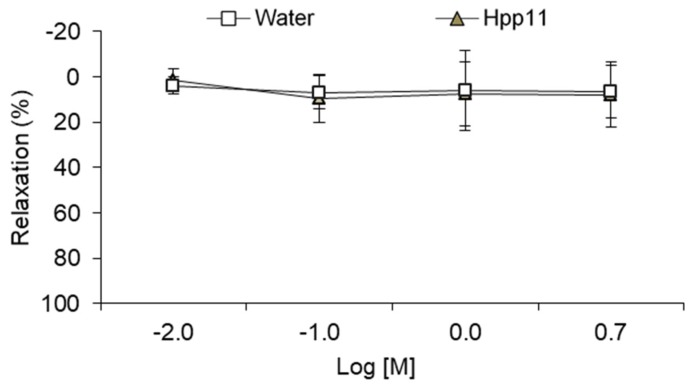
Cumulative concentration–response curves of chicken foot hydrolysate Hpp11 (0.01–5 M) in methoxamine pre-constricted aortic rings from Sprague-Dawley rats. Water was employed as a control, adding the same volume used to carry out the concentration-response curves of Hpp11. Data are mean values ± SEM. Both experimental groups have a minimum of six animals. No significant differences were observed between control group and Hpp11 group.

In addition, to evaluate the existence of other mechanisms of antihypertensive activity in addition to ACE inhibition, the vascular effects of Hpp11 in aorta of SD rats was investigated. As demonstrated in Figure 5, Hpp11 did not produce relaxation in the aortic segments pre-contracted by methoxamine showing a similar effect to the control group.

## 4. Discussion

The beneficial effects of bioactive peptides derived from hydrolyzed dietary proteins have been reported in many studies [33,34]. Some of these peptides demonstrate antihypertensive effects among other activities. Considering that commonly used antihypertensive drugs could present undesirable side effects, the interest in the use of protein hydrolysates to alleviate HTN has increased in recent years [35]. It is well known that chicken proteins are a good source of antihypertensive peptides. Onuh et al. demonstrated that peptides included in chicken skin protein hydrolysates were able to produce an antihypertensive effect after their administration to SHR [22]. Similar results were obtained from Saiga et al., postulating that chicken is a great source of bioactive peptides in part due to its high content of collagen [36], previously demonstrated to be a precursor of bioactive peptides [37]. Considering this, in a previous study our group it was demonstrated that chicken foot proteins are a potential source of antihypertensive peptides [18]. In this previous study, chicken foot hydrolysates were obtained and one of the hydrolysates (Hpp11) exhibited a clear antihypertensive effect when administered at a dose of 5 mL/kg bw to the SHR as well as in vitro ACEI activity [18]. In this sense, protein hydrolysates administered at 100 mg/kg bw have been demonstrated to exert substantial antihypertensive effects [22,28]. Thus, in this study, three different lower doses 85, 55 and 25 mg/kg bw were administered to SHR to identify the dose with the maximum antihypertensive effect. Only the doses of 55 and 85 mg/kg bw exhibited the antihypertensive effect, reaching, in both cases, the maximum decrease at 6 h post-administration. Importantly, no differences between Hpp11 at 55 and 85 mg/kg bw and Captopril 50 mg/kg bw were found, suggesting the potential antihypertensive effect of this hydrolysate. In this sense, many studies reported the beneficial effects of protein hydrolysates in treating HTN conditions [21,22,38,39]; however, it is important to note that the doses used in this study are significantly lower than the doses reported for different protein-derived hydrolysates. Accordingly, the blood pressure-lowering effect exhibited by Hpp11 at 55 and 85 mg/kg bw (−26.33 ± 2.1 and −30.45 ± 1.65 mmHg, respectively) was similar to those reported by Miguel et al. for the antihypertensive hydrolysate from egg white. However, in this study, 100 mg/kg bw dose was required to observe this antihypertensive effect [28]. Interestingly, chicken-leg bone protein hydrolysate showed similar antihypertensive results (−26 mmgHg) being administered at a dose of 50 mg/kg bw [39], demonstrating the antihypertensive potential effect of chicken by-products. Nevertheless, the dose used to obtain this reduction in BP was 600 mg/kg bw [40], significantly higher than the dose of Hpp11 used in the present study. As mentioned before, both doses (55 and 85 mg/kg bw) presented similar antihypertensive effects, but it was only the 55 mg/kg bw dose that maintained the antihypertensive effect 8 h post-administration. The SBP from the Hpp11 treated groups at 55 and 85 mg/kg bw were completely restored 24 h post-administration. It is known that ACE inhibitory activity in vitro does not always correspond to an antihypertensive effect in vivo. This is mainly due to the bioavailability of the ACE inhibitory peptides after oral administration and the fact that peptides may influence blood pressure by mechanisms other than ACE inhibition. In vivo protein digestion could produce peptide modifications that could inactivate or activate antihypertensive peptides [41,42]. Considering this, in vivo assays are always required to demonstrate in vivo bioactivity of these protein hydrolysates able to inhibit ACE in vitro. These observations indicate that the ACE-inhibiting peptides in the 55 and 85 mg/kg bw Hpp11 were bioavailable either intact or in modified forms to exert short-term antihypertensive effects. Then, these peptides were rapidly metabolized into inactive products leading to the subsequent reduction in activity, especially for 85 mg/kg bw Hpp11, with no apparent antihypertensive by 8 h post-administration. A similar antihypertensive pattern was recently described by Udenigwe et al. using by-product hen meat protein hydrolysates [43]. These results showed that at relatively low doses, Hpp11 was able to reduce the SBP in the same manner as Captopril, demonstrating its potential applicability in HTN treatment. Moreover, the fact that relatively low doses are enough to obtain a potent antihypertensive effect increases its industrial value.

It is also important to point out that the administration of Hpp11 to normotensive WKY rats did not modify the BP of these animals. This indicates that the effect of Hpp11 is specific to the hypertensive condition. Therefore, these products could be used as functional foods without any risk in normotensive subjects.

One of the most common mechanisms likely involved in the BP-lowering effect of food peptides is ACE inhibition. Therefore, hydrolysate selection by their ACEI activity in vitro is a potential strategy for the selection of antihypertensive hydrolysates and peptides [6]. In fact, Hpp11 was selected by its great ability to inhibit ACE in vitro. However, in vitro ACEI activity does not always correspond to the same bioactivity in vivo because of the physiological transformations during protein digestion. Thus, plasmatic ACE activity in the SHR treated with 55 mg/kg bw was evaluated 6 h post-administration. ACE activity was significantly reduced in the Hpp11 treated group compared to the water treated group. Similar results were reported after the administration of the ACE-inhibitory peptides contained in egg yolk [44] and soya protein [45].

Moreover, the inhibitory pattern of Hpp11 on in vitro ACE was studied. It was demonstrated that the peptides contained in the hydrolysate bind competitively at the active site of ACE to produce its inhibition. Quirós et al. [46] reported the same inhibition pattern for β-casein-peptides with antihypertensive properties. In this sense, it is well known that the most common mechanism of action of peptides in ACE inhibition is different from that of synthetic drugs. Generally, drugs indiscriminately block ACE and interfere with its activity, while ACE inhibitory peptides competitively block the binding of Ang I to ACE, thereby inhibiting the formation of Ang II [14,47]. Considering our results, Hpp11 produced its in vivo antihypertensive action by inhibiting the ACE activity, which suggests a possible reduction in Ang II release. However, many other systems different from RAAS can contribute to the control of BP. In this context, hydrolysates are complex mixtures of different peptides, and mechanisms of action other than ACE inhibition could be involved in their antihypertensive effect. In fact, it has been reported that antihypertensive food peptides can also have antioxidant, vasodilator, and/or opioid activities. In this sense, Sipola et al. [48] reported on the vasodilator-mediated antihypertensive effect of milk-derived peptides. Similar results were reported by Fujita et al., who reported that the antihypertensive effect of human casein-derived peptides was mediated by inducing relaxation in arteries [49]. To evaluate the potential Hpp11 vasodilator effect, aortic rings from SD rats were pre-contracted with methoxamine and then Hpp11 at increasing doses was administered. To evaluate the potential effects of different compounds on the vasculature, normotensive animals are used. SD rats are normotensive animals, such as WKY. However, SD rats are widely used to perform this type of study, considering that they offer higher response to contractile and relaxing agents than WKY, allowing it to demonstrate more clearly the effect of the tested compound [50].

Nevertheless, Hpp11 did not induce any relaxation in these preparations, showing similar behaviour to the control (water). However, it should be highlighted that SD rats are normotensive animals and Hpp11 may have different effects in the arteries of hypertensive animals. Future studies evaluating the Hpp11 effect on aortic rings from SHR could be performed to analyse the possible differences in their arterial responses to Hpp11. Moreover, the aorta is a conduit artery, and the resistance arteries determine the arterial BP more so than large vessels [51]. Therefore, we also suggest the use of resistance arteries for future studies to evaluate the Hpp11 vasodilator effect.

## 5. Conclusions

In this study, we demonstrated that the most effective antihypertensive dose of the chicken foot hydrolysate Hpp11 was 55 mg/kg after an acute administration. Our results indicate that Hpp11 produces its antihypertensive effect through the inhibition of ACE. Therefore, Hpp11 at low doses could be used as a functional food ingredient with potential therapeutic benefits in the prevention and treatment of HTN. Nevertheless, considering that HTN is a chronic pathology that requires chronic treatment, the evaluation of long-term administration of Hpp11 is necessary in animals and humans before the use of Hpp11 as an antihypertensive functional ingredient. Moreover, further studies are needed to determine the amino acid sequences present in Hpp11, responsible for the observed antihypertensive effect.

## 6. Patents

Patent application “Hydrolysates of chicken leg, their peptides and their uses”: application number P201731065.

## Figures and Tables

**Table 1:** Protein content, humidity, ash content and degree of hydrolysis of the chicken foot hydrolysate Hpp11.

Determinations	(%)
Total protein content ^a^	0.67
Humidity	98.93
Ash content ^b^	0.17
Degree of hydrolysis ^c^	18.85
ACE inhibition ^d^	95.11

^a^ Protein content was estimated by the measure of total nitrogen compounds content measured by the Kjeldahl method, expressed as *w*/*v*; ^b^ Ash content is expressed as g ash/100 g of product; ^c^ Degree of hydrolysis was measured by the TNBS method in which free α-amino groups were determined. The data shown are mean values of each parameter for at least two different hydrolysates assayed under the same conditions. ^d^ Angiotensin-converting enzyme (ACE) inhibitory activity (%).

## References

[1] Messerli F.H., Williams B., Ritz E. (2007). Essential hypertension. Lancet.

[2] Hedayati S., Elsayed E., Reilly R. (2011). Non-pharmacological aspects of blood pressure management: What are the data?. Kidney Int..

[3] Zhuang Y., Sun L., Zhang Y., Liu G. (2012). Antihypertensive effect of long-term oral administration of jellyfish (*Rhopilema esculentum*) collagen peptides on renovascular hypertension. Mar. Drugs.

[4] Cheng F.Y., Wan T.C., Liu Y.T., Chen C.M., Lin L.C., Sakata R. (2009). Determination of angiotensin-I converting enzyme inhibitory peptides in chicken leg bone protein hydrolysate with alcalase. Anim. Sci. J..

[5] Sica D.A. (2004). Angiotensin-converting enzyme inhibitors side effects—Physiologic and non-physiologic considerations. J. Clin. Hypertens..

[6] Margalef M., Bravo F.I., Arola-Arnal A., Muguerza B., Andrade P., Valentao P., Pereira D.M. (2017). Natural angiotensin converting enzyme (ACE) inhibitors with antihypetensive properties. Natural Products Targeting Clinically Relevant Enzymes.

[7] Iwaniak A., Minkiewicz P., Darewicz M. (2014). Food-originating ACE inhibitors, including antihypertensive peptides, as preventive food components in blood pressure reduction. Compr. Rev. Food Sci. Food Saf..

[8] Chakrabarti S., Wu J. (2016). Bioactive peptides on endothelial function. Food Sci. Hum. Wellness.

[9] Fang H., Luo M., Sheng Y., Li Z., Wu Y., Liu C. (2008). The antihypertensive effect of peptides: A novel alternative to drugs?. Peptides.

[10] Martínez-Maqueda D., Miralles B., Recio I., Hernández-Ledesma B. (2012). Antihypertensive peptides from food proteins: A review. Food Funct..

[11] Aleixandre A., Miguel M., Muguerza B. (2008). Péptidos antihipertensivos derivados de proteínas de leche y huevo. Nutr. Hosp..

[12] Huang J., Liu Q., Xue B., Chen L., Wang Y., Ou S., Peng X. (2016). Angiotensin-I-converting enzyme inhibitory activities and in vivo Antihypertensive Effects of Sardine Protein Hydrolysate. J. Food Sci..

[13] Slizyte R., Rommi K., Mozuraityte R., Eck P., Five K., Rustad T. (2016). Bioactivities of fish protein hydrolysates from defatted salmon backbones. Biotechnol. Rep..

[14] Ahhmed A.M., Muguruma M. (2010). A review of meat protein hydrolysates and hypertension. Meat Sci..

[15] Ketnawa S., Rawdkuen S. (2013). Angiotensin converting enzyme inhibitory peptides from aquatic and their processing by-Products: A review. Int. J. Sci. Innov. Discov..

[16] Karamaæ M., Flaczyk E., Wanasundara P.K.J.P.D., Amarowicz R. (2005). Angiotensin I-converting enzyme (ACE) inhibitory activity of hydrolysates obtained from muscle food industry by-products—A short report. Pol. J. Food Nutr. Sci..

[17] Mora L., Reig M., Toldrá F. (2014). Bioactive peptides generated from meat industry by-products. Food Res. Int..

[18] Bravo F.I., Arola L., Muguerza B. (2017). Procedure for obtaining a hydrolysate claw chicken leg with antihypertensive activity, and peptides obtained hydrolysate containing. Patent.

[19] Lasekan A., Abu Bakar F., Hashim D. (2013). Potential of chicken by-products as sources of useful biological resources. Waste Manag..

[20] Toldrá F., Aristoy M.C., Mora L., Reig M. (2012). Innovations in value-addition of edible meat by-products. Meat Sci..

[21] Onuh J.O., Girgih A.T., Aluko R.E., Aliani M. (2013). Inhibitions of renin and angiotensin converting enzyme activities by enzymatic chicken skin protein hydrolysates. Food Res. Int..

[22] Onuh J.O., Girgih A.T., Malomo S.A., Aluko R.E., Aliani M. (2015). Kinetics of in vitro renin and angiotensin converting enzyme inhibition by chicken skin protein hydrolysates and their blood pressure lowering effects in spontaneously hypertensive rats. J. Funct. Foods.

[23] Onuh J.O., Girgih A.T., Nwachukwu I., Ievari-Shariati S., Raj P., Netticadan T., Aluko R.E., Aliani M. (2016). A metabolomics approach for investigating urinary and plasma changes in spontaneously hypertensive rats (SHR) fed with chicken skin protein hydrolysates diets. J. Funct. Foods.

[24] Majumder K., Wu J. (2015). Molecular Targets of Antihypertensive Peptides: Understanding the mechanisms of action based on the pathophysiology of hypertension. Int. J. Mol. Sci..

[25] Miguel M., Manso M., Aleixandre A., Alonso M.J., Salaices M., Lopez-Fandino R. (2007). Vascular effects, angiotensin I-converting enzyme (ACE)-inhibitory activity, and antihypertensive properties of peptides derived from egg white. J. Agric. Food Chem..

[26] Girgih A.T., Nwachukwu I.D., Onuh J.O., Malomo S.A., Aluko R.E. (2016). Antihypertensive properties of a pea protein hydrolysate during short- and long-term oral administration to spontaneously hypertensive rats. J. Food Sci..

[27] Lin H.-C., Alashi A.M., Aluko R.E., Sun Pan B., Chang Y.-W. (2017). Antihypertensive properties of tilapia (*Oreochromis* spp.) frame and skin enzymatic protein hydrolysates. Food Nutr. Res..

[28] Miguel M., Alonso M.J., Salaices M., Aleixandre A., López-Fandiño R. (2007). Antihypertensive, ACE-inhibitory and vasodilator properties of an egg white hydrolysate: Effect of a simulated intestinal digestion. Food Chem..

[29] Association of Official Analytical Chemists (AOAC) (1995). Official Methods of Analysis.

[30] Adler-Nissen J. (1979). Determination of the degree of hydrolysis of food protein hydrolysates by trinitrobenzenesulfonic acid. J. Agric. Food Chem..

[31] Quirós A., del Contreras M.M., Ramos M., Amigo L., Recio I. (2009). Stability to gastrointestinal enzymes and structure–activity relationship of β-casein-peptides with antihypertensive properties. Peptides.

[32] Buñag R.D. (1973). Validation in awake rats of a tail-cuff method for measuring systolic pressure. J. Appl. Physiol..

[33] Yamamoto N. (1997). Antihypertensive peptides derived from food proteins. Biopolym. Pept. Sci. Sect..

[34] Udenigwe C.C., Mohan A. (2014). Mechanisms of food protein-derived antihypertensive peptides other than ACE inhibition. J. Funct. Foods.

[35] Hou Y., Wu Z., Dai Z., Wang G., Wu G. (2017). Protein hydrolysates in animal nutrition: Industrial production, bioactive peptides, and functional significance. J. Anim. Sci. Biotechnol..

[36] Saiga A., Iwai K., Hayakawa T., Takahata Y., Kitamura S., Nishimura T., Morimatsu F. (2008). Angiotensin I-converting enzyme-inhibitory peptides obtained from chicken collagen hydrolysate. J. Agric. Food Chem..

[37] Fu Y., Therkildsen M., Aluko R.E., Lametsch R. (2018). Exploration of collagen recovered from animal by-products as a precursor of bioactive peptides: Successes and challenges. Crit. Rev. Food Sci. Nutr..

[38] Balti R., Bougatef A., Ali N.E.H., Zekri D., Barkia A., Nasri M. (2010). Influence of degree of hydrolysis on functional properties and angiotensin I-converting enzyme-inhibitory activity of protein hydrolysates from cuttlefish (*Sepia officinalis*) by-products. J. Sci. Food Agric..

[39] Cheng F.Y., Wan T.C., Liu Y.T., Lai K.M., Lin L.C., Sakata R. (2008). A study of in vivo antihypertensive properties of enzymatic hydrolysate from chicken leg bone protein. Anim. Sci. J..

[40] Li G.-H., Qu M.-R., Wan J.-Z., You J.-M. (2007). Antihypertensive effect of rice protein hydrolysate with in vitro angiotensin I-converting enzyme inhibitory activity in spontaneously hypertensive rats. Asia Pac. J. Clin. Nutr..

[41] Vermeirssen V., Van Camp J., Verstraete W. (2004). Bioavailability of angiotensin I converting enzyme inhibitory peptides. Br. J. Nutr..

[42] Jao C.-L., Huang S.-L., Hsu K.-C. (2012). Angiotensin I-converting enzyme inhibitory peptides: Inhibition mode, bioavailability, and antihypertensive effects. BioMedicine.

[43] Udenigwe C.C., Girgih A.T., Mohan A., Gong M., Malomo S.A., Aluko R.E. (2017). Antihypertensive and bovine plasma oxidation-inhibitory activities of spent hen meat protein hydrolysates. J. Food Biochem..

[44] Yoshii H., Tachi N., Ohba R., Sakamura O., Takeyama H., Itani T. (2001). Antihypertensive effect of ACE inhibitory oligopeptides from chicken egg yolks. Comp. Biochem. Physiol. C.

[45] Yang H.-Y., Yang S.-C., Chen J.-R., Tzeng Y.-H., Han B.-C. (2004). Soyabean protein hydrolysate prevents the development of hypertension in spontaneously hypertensive rats. Br. J. Nutr..

[46] Quirós A., Ramos M., Muguerza B., Delgado M.A., Miguel M., Aleixandre A., Recio I. (2007). Identification of novel antihypertensive peptides in milk fermented with *Enterococcus faecalis*. Int. Dairy J..

[47] Alderman C.P. (1996). Adverse effects of the angiotensin-converting enzyme inhibitors. Ann Pharmacoter..

[48] Sipola M., Finckenberg P., Vapaatalo H., Pihlanto-Leppälä A., Korhonen H., Korpela R., Nurminen M.-L. (2002). Alpha-lactorphin and beta-lactorphin improve arterial function in spontaneously hypertensive rats. Life Sci..

[49] Fujita H., Suganuma H., Usui H., Kurahashi K., Nakagiri R., Sasaki R., Yoshikawa M. (1996). Vasorelaxation by casomokinin L, a derivative of β-casomorphin and casoxin D, is mediated by NK1receptor. Peptides.

[50] Rahmani M.A., DeGray G., David V., Ampy F.R., Jones L. (1999). Comparison of calcium import as a function of contraction in the aortic smooth muscle of Sprague-Dawley, Wistar Kyoto and spontaneously hypertensive rats. Front. Biosci..

[51] Pons Z., Arola L. (2014). Involvement of nitric oxide and prostacyclin in the antihypertensive effect of low-molecular-weight procyanidin rich grape seed extract in male spontaneously hypertensive rats. J. Funct. Foods.

